# Inhibition of PI3K/Akt/mTOR signaling pathway alleviates ovarian cancer chemoresistance through reversing epithelial-mesenchymal transition and decreasing cancer stem cell marker expression

**DOI:** 10.1186/s12885-019-5824-9

**Published:** 2019-06-24

**Authors:** Junli Deng, Xupeng Bai, Xiaojie Feng, Jie Ni, Julia Beretov, Peter Graham, Yong Li

**Affiliations:** 1 Cancer Care Centre, St George Hospital, 4-10 South St, Kogarah, NSW 2217 Australia; 2 St George and Sutherland Clinical School, UNSW Sydney, Kensington, NSW 2052 Australia; 3 Department of Gynaecological Oncology, Henan Cancer Hospital, Henan, 450008 China; 4 Anatomical Pathology, NSW Health Pathology, St. George Hospital, Kogarah, NSW 2217 Australia; 5 School of Basic Medical Sciences, Zhengzhou University, Henan, 450001 China

**Keywords:** Ovarian cancer, Chemoresistance, EMT, CSC, PI3K/Akt/mTOR signaling

## Abstract

**Background:**

Ovarian cancer is the most common malignant tumor of the female reproductive tract. Chemoresistance is a major challenge for current ovarian cancer therapy. However, the mechanism underlying epithelial ovarian cancer (EOC) chemoresistance is not completely uncovered. The phosphatidylinositol-3-kinase (PI3K)/Akt/mammalian target of rapamycin (mTOR) signaling is an important intracellular pathway in regulating cell cycle, quiescence, and proliferation. The aim of this study is to investigate the role of PI3K/Akt/mTOR signaling pathway and its association with epithelial-mesenchymal transition (EMT) and cancer stem cell (CSC) marker expression in EOC chemoresistance.

**Methods:**

The expressions of EMT and CSC markers were detected by immunofluorescence, western blot, and quantitative real-time PCR. BEZ235, a dual PI3K/mTOR inhibitor, was employed to investigate the role of PI3K/Akt/ mTOR signaling in regulating EMT and CSC marker expression. Students’ t test and one-way ANOVA with Tukey’s post-hoc test were used to compare the data from different groups.

**Results:**

We found that EMT and CSC marker expression were significantly enhanced in chemoresistant EOC cells, which was accompanied by the activation of PI3K/Akt/mTOR signaling. Compared with single cisplatin treatment, combined treatment with BEZ235 and cisplatin significantly disrupted the colony formation ability, induced higher ROS level and more apoptosis in chemoresistant EOC cells. Furthermore, the combination approach effectively inhibited PI3K/Akt/mTOR signaling pathway, reversed EMT, and decreased CSC marker expression in chemoresistant EOC cells compared with cisplatin mono-treatment.

**Conclusions:**

Our results first demonstrate that EMT and enhanced CSC marker expression triggered by activated PI3K/Akt/mTOR signaling are involved in the chemoresistance of EOC, and BEZ235 in combination with cisplatin might be a promising treatment option to reverse EOC chemoresistance.

**Electronic supplementary material:**

The online version of this article (10.1186/s12885-019-5824-9) contains supplementary material, which is available to authorized users.

## Background

Ovarian cancer is the most common malignant tumor of the female reproductive tract. It was reported to occur in 22,240 women and caused 14,080 deaths in 2017 in the world [1]. Around 30 different types of ovarian cancer are defined based on the cell origins, such as epithelial ovarian cancer (EOC), germ cell ovarian cancer, and stromal cell ovarian cancer. Thereinto, EOC is the most common type and accounts for approximately 90% of ovarian malignancy [2].

Chemoresistance is a major challenge for current ovarian cancer therapy. The mechanism of EOC chemoresistance is not completely uncovered. Studies from different groups indicated that epithelial-mesenchymal transition (EMT) and cancer stem cell (CSC) were closely associated with the chemoresistance, metastasis and tumor relapse in EOC patients [3]. EMT is a biological process in which the phenotype of epithelial cells is transformed into a mesenchymal phenotype by specific procedures [4]. During EMT, cells downregulate the epithelial cellular adhesion markers and gain mesenchymal properties by increasing the expression of mesenchymal markers [5, 6]. Recently, a close association between EMT-like cancer cells and chemotherapeutic resistance has been revealed [7, 8]. However, the role of EMT in EOC chemoresistance is not fully elucidated.

On the other hand, mounting evidence suggests that CSCs are the culprits of therapeutic resistance and cancer metastasis, leading to the tumor relapse and death in patients [9]. CSCs are capable of self-renewal, recapitulating the heterogeneity of the tumors, and differentiating into a new tumor bulk in the appropriate microenvironment. K Abubaker, et al. [10] found that surviving ovarian cancer cells in mice xenograft after chemotherapy were enriched with CSC phenotypes [85]. Furthermore, IA Silva, et al. [11] reported that ALDH^+^CD133^+^ CSC-like cells within human primary ovarian tumors displayed enhanced chemoresistant phenotypes as compared to the parental cells. Also, the presence of these cells in primary ovarian tumors was associated with a shorter disease-free survival and overall survival in patients [11]. These studies suggest that CSCs are closely associated with ovarian cancer chemoresistance.

Phosphatidylinositol-3-kinase (PI3K)/Akt/mammalian target of rapamycin (mTOR) signaling is an important intracellular pathway in regulating cell cycle, quiescence, and proliferation. Various somatic mutations in phosphatase and tensin homolog (PTEN), Akt1, and mTOR that induce enhanced PI3K/Akt/mTOR signaling have been found in ovarian cancer [12–16]. The hyperactivation of PI3K/Akt/mTOR signaling was implicated in cancer metastasis and chemoresistance [17–19]. It was reported that PI3K regulated G1 cell cycle and apoptosis in ovarian cancer by activating Akt/mTOR/p70S6K1 signaling [20], and inhibition of PI3K can disrupt ovarian cancer cell proliferation and trigger cell death. More importantly, the inhibition of PI3K/Akt/mTOR signaling was found to re-sensitize chemoresistant ovarian cancer cells to chemotherapeutic drugs [21, 22]. These findings establish PI3K/Akt/mTOR signaling as an attractive therapeutic target for ovarian cancer treatment. However, the correlation between PI3K/Akt/mTOR signaling and EMT or CSC in EOC chemoresistance remains unclear.

In the current study, we found that the acquisition of EMT and enhancement of CSC marker expression in chemoresistant EOC cells were associated with the activation of PI3K/Akt/mTOR signaling. More importantly, BEZ235 in combination with cisplatin can significantly reduce the expressions of EMT and CSC markers and restore the sensitivity of chemoresistant EOC cells to cisplatin. These results may provide further evidence for better understanding the mechanism underlying EOC chemoresistance and evaluating the effect of PI3K inhibition in EOC chemosensitization.

## Methods

### Antibodies and reagents

Antibodies used for this study are summarized in Additional file 3: Table S1. Cisplatin, carboplatin, paclitaxel were obtained from Sigma-Aldrich (NSW, Australia), while BEZ235 (dactolisib) was obtained from Jomar Life Research (Melbourne, VIC, Australia). BEZ235 stock solution was prepared at 1 mg/mL in N-methyl-2-pyrrolidone + polyethylene glycol 300 (1:9, v/v), while other drug stock solutions were prepared in dimethyl sulfoxide (DMSO, Sigma–Aldrich). All working solutions were diluted using cell culture medium with the final concentration of organic solvent lower than 0.1%.

### Cell culture

A2780 and A2780-cis EOC cell lines were obtained from Sigma-Aldrich (Castle Hill, NSW, Australia). The development of A2780-cis cell line is described in https://www.sigmaaldrich.com/catalog/product/sigma/cb_93112517. IGROV1 cell line was obtained from the American Type Culture Collection (ATCC) (Rockville, MD, USA), while IGROV1-cis EOC cell line was kindly provided by Prof. Jan H.M. Schellens (Netherlands Cancer Institute, AMS, Netherland). The development of IGROV1-cis cell line is described in a previous publication [23]. All cell culture reagents were purchased from ThermoFisher Scientific (Melbourne, VIC, Australia). All cell lines were cultured in RPMI-1640 medium supplemented with 10% fetal bovine serum (FBS), 50 units/mL penicillin, and 50 μg/mL streptomycin. 1 μM and 3.3 μM cisplatin were added to the medium every 2–3 passages for A2780-cis and IGROV1-cis cell lines, respectively. All cell lines were maintained in a humidified incubator at 37 °C and 5% CO_2_.

### Proliferation assay

1 × 10^3^ cells were seeded in 96-well plates and cultured for consecutive 7 days at 37 °C and 5% CO_2_. The cell number was detected using CyQUANT® Cell Proliferation Assay Kit (Life Technologies, Scoresby, VIC, Australia) according to the protocol. The fluorescence intensity was measured using a microplate reader (Bio-Tec, Hercules, CA, USA) with excitation at ~ 485 nm and emission at ~ 530 nm.

### Cell viability detection

Cell viability detection was performed using MTT assay. Briefly, 8 × 10^3^ cells were seeded in 96-well plates for 24 h and then treated with a range of concentrations of drugs. After 48-h incubation, the cell viability was detected using 3-(4,5-dimethylthiazol-2-yl)-2,5-diphenyltetrazolium bromide (0.5 mg/mL) and Bio-Tec microplate reader at 562 nm wavelength. The cell viability curves were generated by Prism 7 (GraphPad, San Diego, CA, USA) and IC_50_ values were calculated.

### Matrigel invasion assay

The invasion ability of A2780, A2780-cis, IGROV1 and IGROV1-cis cells was evaluated by Matrigel Transwell® plates (BD Bioscience, NSW, Australia) according to manufacturer’s specification. The invasion ability was calculated as follows: invasion ratio = [(average number of cells invading through matrigel insert) / (average number of cells migrating through control insert)] × 100%.

### Wound-healing assay

Cell migration ability was evaluated by wound-healing assay. Briefly, 3 × 10^5^ cells were seeded in 6-well plates for 24 h. Monolayer cells were scraped using a sterile 200 μL tip, which caused a straight scratch (wound). The wounded monolayer cells were washed to remove cell debris and then replenished with fresh cell culture medium. Representative images were obtained after 0 h, 48 h, 96 h at 40x magnification using a light microscope.

### Cell cycle analysis

Cell cycle analysis was conducted using flow cytometry. Briefly, the sample containing trypsinized adherent, and floating cells with 1 μL FxCycle Violet stain was analyzed at 405-nm excitation using FACSCanto II Flow Cytometer (BD Biosciences, San Jose, CA, USA), and emission was collected with a 450/50 band-pass filter. The results further were analyzed using FlowJo V10 software (Tree Star Inc., Ashland, OR, USA) to determine cell cycle distribution.

### Immunofluorescence

Cells grown on glass cover-slips (1 × 10^5^ cells) were fixed by methanol for 10 mins at RT, incubated with various primary antibodies (Additional file 3: Table S1) and then Alexa Fluro-488 goat anti-rabbit IgG, Alexa Fluro-488 goat anti-mouse IgG, or Alexa Fluro-488 donkey anti-goat IgG. Propidium iodide (PI) (0.2 mg/L) was used for nuclei staining. The staining results were visualized using FV300/FV500 laser scanning confocal microscope (Olympus, Tokyo, Japan). The mean intensity of immunostaining from three randomly selected fields was scored as 0 (negative, < 25%), 1 (weak, 25–50%), 2 (moderate, 50–70%), and 3 (strong, > 75%) by two observers (J.B. and Y.L.).

### Quantitative real-time PCR (qRT-PCR)

Primers for EMT (E-cadherin, N-cadherin, Vimentin), CSC (CD44v6, CD117, ALDH1A1, Snail) markers, and GAPDH (control) were synthesized by Invitrogen (Mulgrave, VIC, Australia). Samples were analyzed as previously described [24]. The values were obtained as the threshold cycle for each gene and normalized to the reference gene (GAPDH). The 2^-ΔΔCT^ method was used to calculate relative changes for each gene in different cell lines.

### Western blot

Protein expressions were examined using western blot as previously described [25]. The membrane proteins were incubated with different primary antibodies (Additional file 3: Table S1), followed by incubation with HRP-conjugated secondary antibodies. Immunoreactive bands were visualized using ImageQuant LAS4000 system (GE Healthcare, USA).

### Colony formation assay

1.5 × 10^3^ cells were seeded in 10-cm dishes for 48 h at 37 °C and 5% CO_2_ and then treated with vehicle control, ½ IC_50_ BEZ235, ½ IC_50_ cisplatin, or combination treatment of ½ IC_50_ BEZ235 and ½ IC_50_ cisplatin, respectively. After 2-day treatment, the drug-containing medium was replaced with fresh media and all cells were incubated for an additional 10 days until colonies were large enough to be clearly distinguished. The colonies, defined as groups of > 50 cells, were counted manually with the aid of INT2 inverted microscope (Olympus, Tokyo, Japan).

### Apoptosis detection

Cell apoptosis was detected using AO/EB assay. 2 × 10^5^ A2780-cis or IGROV1-cis cells were treated with vehicle control, ½ IC_50_ BEZ235, ½ IC_50_ cisplatin, or combination treatment of ½ IC_50_ BEZ235 and ½ IC_50_ cisplatin for 24 h, respectively. Cells were then stained with AO/EB (Sigma-Aldrich) according to manufacturer’s protocol and examined by FV300/FV500 laser scanning confocal microscope (Olympus, Tokyo, Japan) at 200x magnification. Apoptotic cells were characterized by nuclear condensation and fragmentation.

### Reactive oxygen species (ROS) assay

2 × 10^5^ A2780-cis and IGROV1-cis cells were treated with vehicle control, ½ IC_50_ BEZ235, ½ IC_50_ cisplatin, or combination treatment of ½ IC_50_ BEZ235 and ½ IC_50_ cisplatin, respectively. After treatments for 48 h, the cells were detached, centrifuged, and re-suspended in 1× PBS. CellROX® Green Reagent (Life Technologies, Scoresby, VIC, Australia) was added to the cells to a final concentration of 5 μM and incubated at 37 °C for 30 mins according to the manufacturer’s protocol. The fluorescence was detected by FACSCanto II Flow Cytometer (BD Biosciences, San Jose, CA, USA) with excitation at ~ 485 nm and emission at ~ 520 nm.

### Statistical analysis

All experiments were repeated in triplicate. The data were expressed as mean ± standard deviation (SD) for continuous variables while frequencies (%) for categorical variables. Students’ t test and one-way ANOVA with post-hoc Tukey’s test were used to compare the data from different groups. *P* < 0.05 was considered statistically significant.

## Results

### EOC-cis cell lines showed enhanced resistance to cisplatin and enhanced metastatic potential

**Fig. 1:**
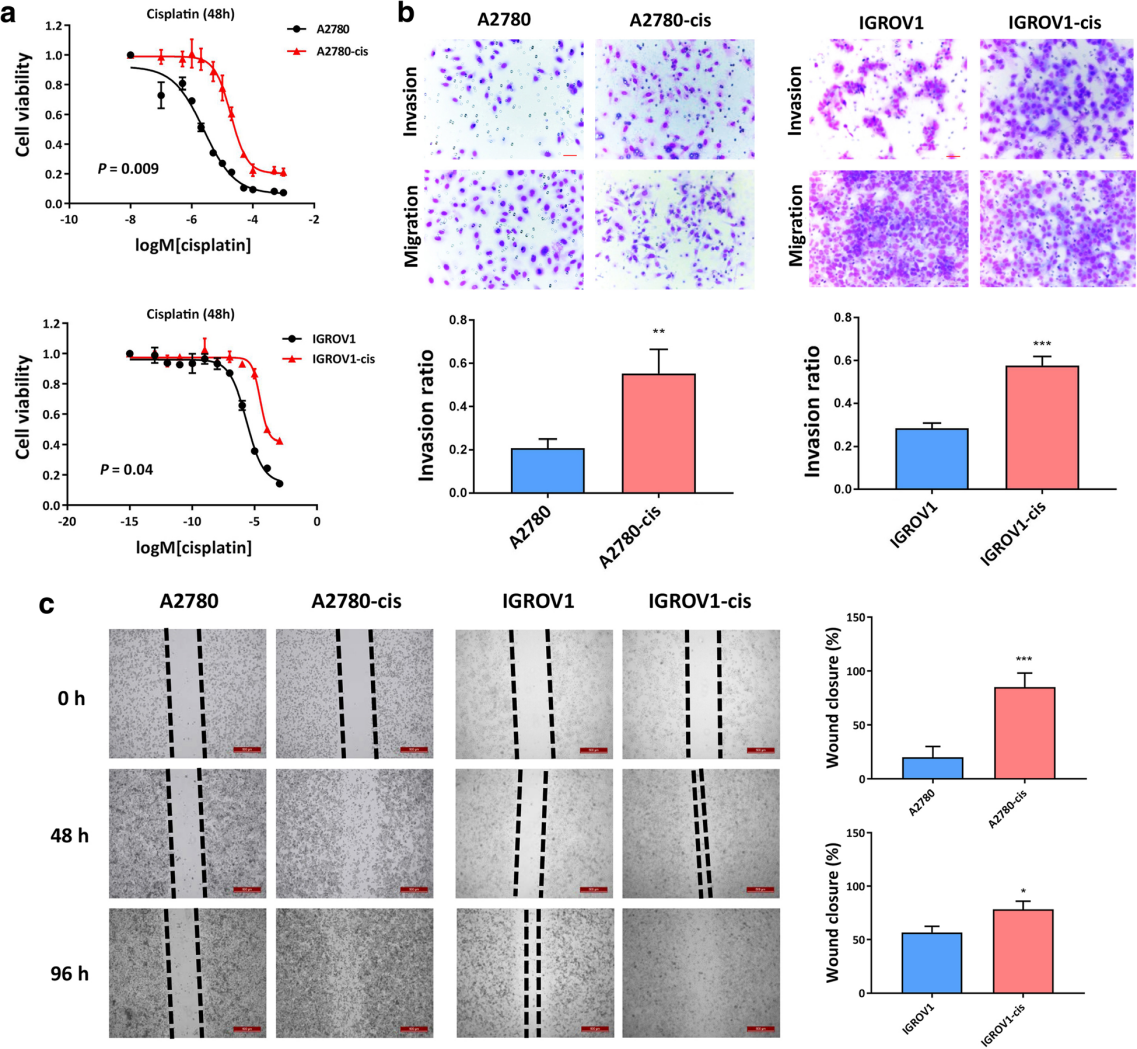
EOC-cis cell lines showed enhanced resistance to cisplatin and enhanced metastatic potential. **a** Cells were treated with different concentrations of cisplatin for 48 h and cell viability was tested using cell proliferation assay. EOC-cis cells showed significant resistance to cisplatin. **b** The invasion ability of cells within 48 h was detected using Matrigel invasion assay. Representative images for EOC cell invasion and migration were photogrphed at 200 x magnification. The invasive potential of EOC-cis cell lines was significantly increased as compared to parental cell lines. **c** The migration ability of cells within 96 h was detected by wound-healing assay. Typical images were obtained at 40x amplification at 0 h, 48 h, and 96 h and the percentage of wound closure area in 96 h was compared. A2780-cis and IGROV1-cis had a higher wound closure rate than the corresponding parental cells. All data were expressed as mean ± SD. ^**^P < 0.01 and ^***^P < 0.001 versus parental cells (*n* = 3)

Cell proliferation results showed that EOC-cis cells (A2780-cis and IGROV-1-cis) grew at a lower proliferation rate compared with their parental cells (Additional file 1: Figure S1a). Also, EOC-cis cells displayed a more quiescent status with more cells staying in G0/G1 phase as compared to their parental cells (Additional file 1: Figure S1b). To confirm the chemoresistance of two EOC-cis cell lines, the cytotoxic assay was performed in the presence of increased concentrations of cisplatin (Additional file 3: Table S2). The results showed that EOC-cis cells demonstrated significantly enhanced resistance to cisplatin **(**Fig. 1a**)**. In addition, A2780-cis cells showed significant cross-resistance to carboplatin, while IGROV1-cis cells showed cross-resistance to both carboplatin and paclitaxel **(**Additional file 2: Figure S2). The IC_50_ values of each drug for cell line within 48 h are summarized in Additional file 3: Table S3. The fold changes of IC_50_ values of cisplatin, carboplatin, and paclitaxel in A2780-cis cells were about 10, 16, and 4, respectively, compared with A2780, and approximately 34, 34, and 156, respectively, in IGROV1-cis cells compared with IGROV1.

Furthermore, EOC-cis cells also showed substantially increased metastatic potential as compared to the parental cells (Fig. 1b). The average invasion rate for A2780-cis and IGROV1-cis was 55 and 58%, respectively, while the average invasion rate for A2780 and IGROV1 was only 21 and 28%, respectively **(**Fig. 1b**)**. Results from the wound-healing assay showed that A2780-cis and IGROV1-cis had a significantly higher wound closure rate in 96 h compared with their sensitive parental cells (Fig. 1c). These results suggest that EOC-cis cells have higher metastatic potential.

### EOC-cis cells showed higher expressions of EMT and CSC markers

**Fig. 2:**
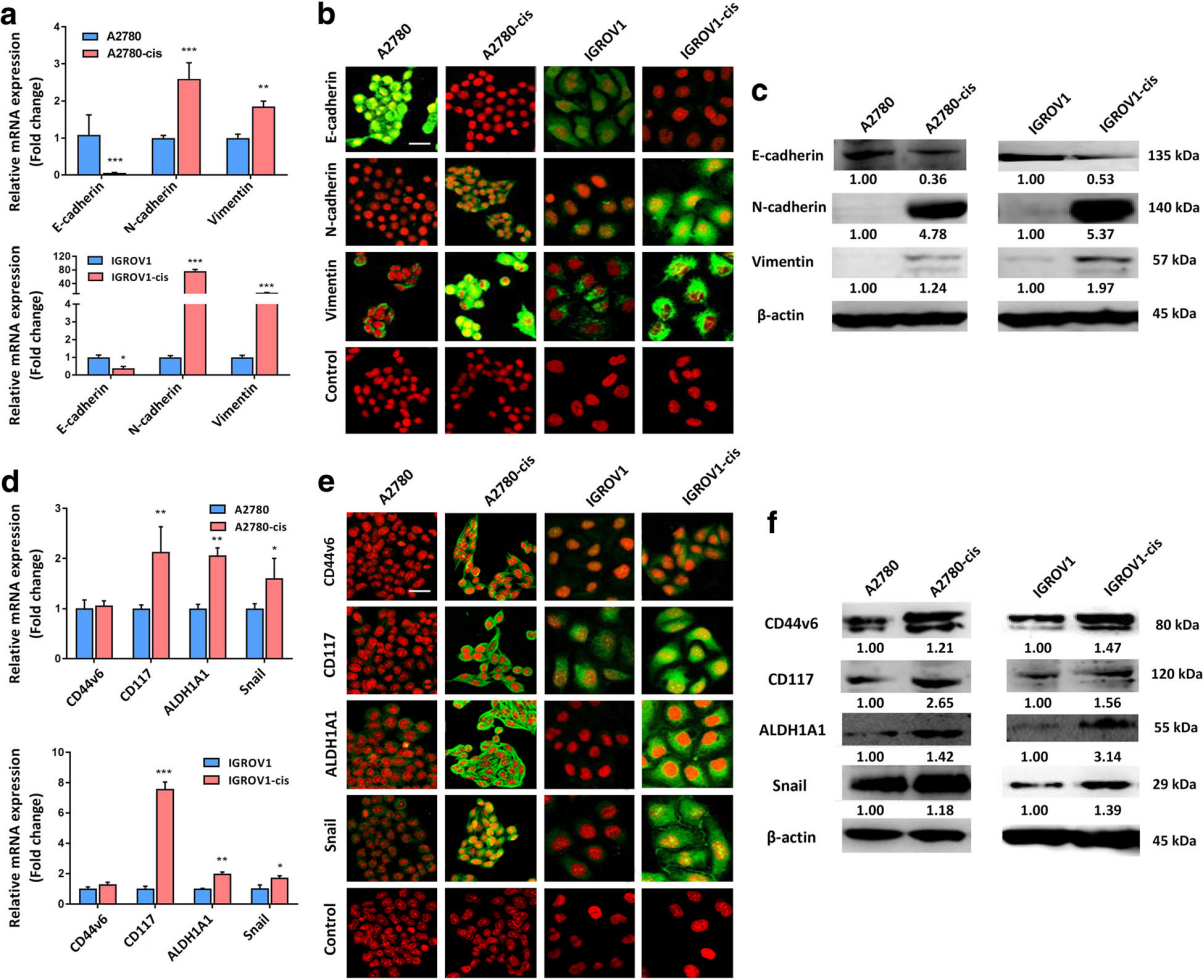
EOC-cis cells showed higher expressions of EMT and CSC markers. **a** mRNA expressions of EMT markers were detected by qRT-PCR. GAPDH was used as a control. **b** Typical images of immnuflourescence for the expressions of EMT markers in EOC-cis and parental cells were obtained at 200x magnification (Green). Red indicates nuclei stain. **c** Protein expressions of EMT markers were detected by western blot. β-actin was used as a loading control. **d** mRNA expressions of CSC markers in EOC-cis cell lines were detected by qRT-PCR. GAPDH was used as a control. **e** Typical images of immnuflourescence for the expressions of CSC markers in EOC-cis and parental cells were obtained at 200x magnification (Green). Red indicates nuclei stain. **f** Protein expressions of CSC markers were detected by western blot. β-actin was used as a loading control. All data were expressed as mean ± SD. ^*^P < 0.05, ^**^P < 0.01, and ^***^P < 0.001 versus parental cells (*n* = 3)

As EOC-cis cell lines showed significantly increased invasion and migration ability, we then investigated whether this is associated with EMT. The results from qRT-PCR showed that the expressions of mesenchymal markers, including N-cadherin and Vimentin, were significantly increased in EOC-cis cell lines, while the expression of epithelial marker E-cadherin was decreased (Fig. 2a). Consistently, immunofluorescence images also showed significantly decreased E-cadherin and markedly increased N-cadherin and Vimentin in EOC-cis cell lines compared with parental cells (Fig. 2b). The immunofluorescence staining scores are summarized in Additional file 3: Table S4. The results were also further confirmed by western blot analysis (Fig. 2c).

It was reported that EMT was associated with an enhanced stemness [3]. Thus, we also detected the expression difference of several ovarian cancer stem cell markers, including CD44v6, CD117, aldehyde dehydrogenase 1 family member A1 (ALDH1A1), and Snail, between EOC-cis and parental cells. Results from qRT-PCR showed that the expressions of CD117, ALDH1A1, and Snail were significantly increased in EOC-cis cell lines (Fig. 2d). Immunofluorescence images showed the higher expressions of CD44v6, CD117, ALDH1A1, and Snail in two EOC-cis cell lines compared with their parental cell lines (Fig. 2e). Negative to weak staining was observed in parental cells (Fig. 2e). CSC marker immunostaining scores are shown in Additional file 3: Table S5. Results from western blot were consistent with the immunofluorescence (Fig. 2f). These results indicate that EOC-cis cells are characterized by enhanced EMT and CSC marker expression.

### PI3K/Akt/mTOR signaling was activated in EOC-cis cells and BEZ235 can sensitize EOC-cis cells to cisplatin

**Fig. 3:**
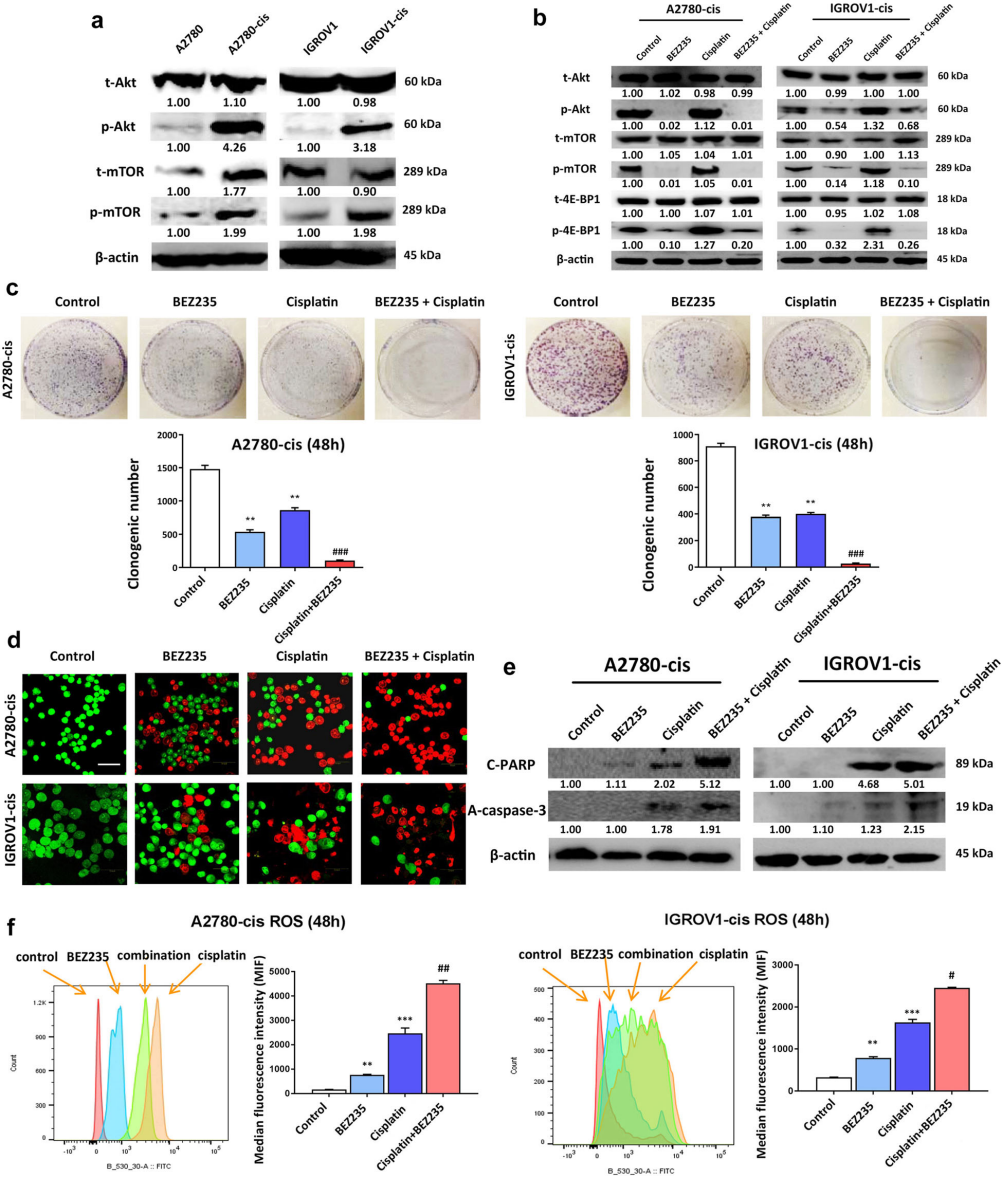
PI3K/Akt/mTOR signaling was activated in EOC-cis cells and BEZ235 can sensitize EOC-cis cells to cisplatin. **a** The protein expressions of Akt and mTOR were detected by western blot. β-actin was used as a loading control. **b** Combination treatment of BEZ235 and cisplatin significantly reduced the expressions of PI3K/Akt/mTOR signaling pathway proteins (p-AKT, pmTOR and p-4E-BP1) as compared to cisplatin mono-treatment group. **c** Colony formation ability of EOC-cis cell lines was detected after treatment with BEZ235 (½ IC_50_), cisplatin (½ IC_50_), or in combination for 48 h. Typical images of colony for different treatment groups are shown. **d** The apoptosis of EOC-cis cells after different treatments was tested with AO/EB assay. Red indicates cells undergoing apoptosis, while green indicates normal cells. Photos were obtained at 200x magnification. **e** The expressions of pre-apoptotic proteins in EOC-cis cells after different treatments were detected by western blot. β-actin was used as a loading control. **f** Quantitation analysis of ROS was performed using flow cytometry with median fluorescence intensity (MFI) detection. Fluorescence emission spectra of ROS in control and treatment groups of EOC-cis cells was shown. ROS level was significantly increased in the combination treatment group compared with single cisplatin treatment in both EOC-cis cell lines. All data were expressed as mean ± SD. ^**^P < 0.01 and ^***^P < 0.01 versus control group, while ^#^*P* < 0.05, ^##^*P* < 0.01, and ^###^*P* < 0.001 versus cisplatin treatment group (*n* = 3)

Involvement of PI3K/Akt/mTOR pathway in EOC chemoresistance was investigated in this study using western blot analysis. The results showed that both p-Akt and p-mTOR were up-regulated in EOC-cis cells, while the increased expression of t-mTOR was only observed in A2780-cis cells (Fig. 3a), suggesting that PI3K/Akt/mTOR signaling was activated in EOC-cis cells. To investigate whether PI3K inhibition can sensitize EOC-cis cells to cisplatin, a dual PI3K and mTOR inhibitor, BEZ235, was employed for the following study. As shown in Fig. 3b, the expressions of p-Akt, p-mTOR and phosphorylated 4E-binding protein 1 (p-4EBP1) were significantly decreased in EOC-cis cells treated with the combination of BEZ235 and cisplatin as compared to cisplatin mono-treatment group. Furthermore, the representative images for colony formation after different treatments are shown in Fig. 3c. The results showed that combination treatment of BEZ235 (½ IC_50_) and cisplatin (½ IC_50_) could effectively reduce the clonogenic number in two EOC-cis cell lines compared with the single treatment group (Fig. 3c), suggesting that PI3K/mTOR inhibition can increase the chemosensitivity of EOC-cis cells. Additionally, the increased apoptosis was also confirmed using AO/EB assay. The results showed that the percentage of cells undergoing apoptosis was obviously increased in the combination treatment group (Fig. 3d). Consistently, the protein expressions of two pre-apoptotic markers, cleaved poly-ADP-ribose polymerase (c-PARP) and active caspase 3 (a-caspase-3), were significantly increased in the combination treatment group as compared to cisplatin mono-treatment group (Fig. 3e). Along with increased apoptosis, the level of reactive oxygen species (ROS) in EOC-cis cells was also markedly up-regulated by the combination treatment of BEZ235 and cisplatin compared with the single treatment (Fig. 3f). The significantly increased median fluorescence intensity of ROS in EOC-cis cells are shown in Fig. 3f, indicating that ROS was further induced by the combination treatment, leading to the sensitizing effect of PI3K inhibition.

### Combination treatment of BEZ235 and cisplatin inhibited EMT and decreased CSC marker expression

**Fig. 4:**
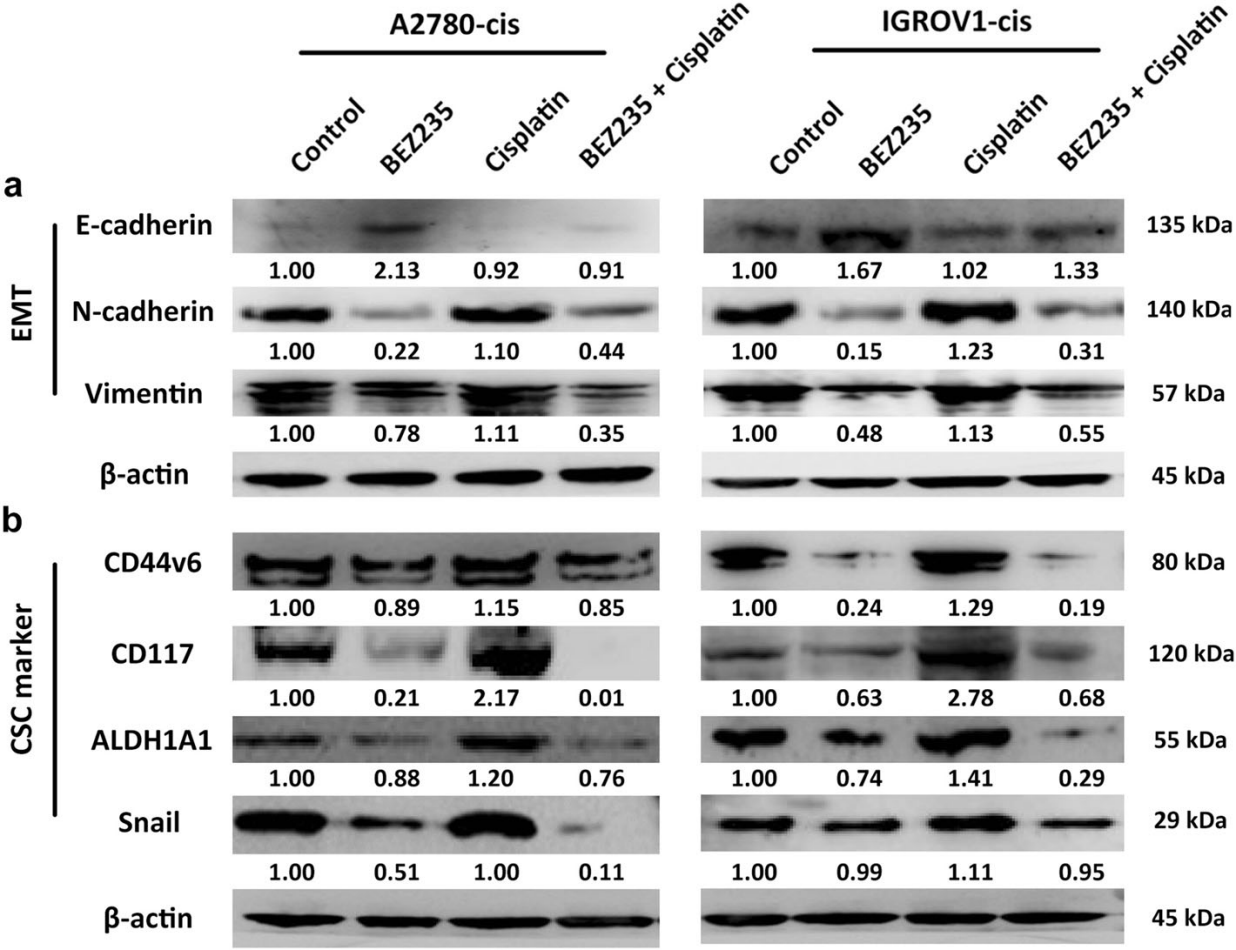
Combination treatment of BEZ235 and cisplatin inhibited EMT and decreased CSC marker expression. **a** The protein expressions of EMT markers in EOC-cis cells after treatment with BEZ235 (½ IC_50_), cisplatin (½ IC_50_), or in combination for 48 h were detected by western blot. β-actin was used as a loading control. Combination treatment of BEZ235 and cisplatin obviously reversed EMT in EOC-cis cells. **b** The protein expressions of CSC markers in EOC-cis cells after treatment with BEZ235 (½ IC50), cisplatin (½ IC50), or in combination for 48 h were detected by western blot. β-actin was used as a loading control. Combination treatment of BEZ235 and cisplatin markedly inhibited CSC marker expression in EOC-cis cells

**Fig. 5:**
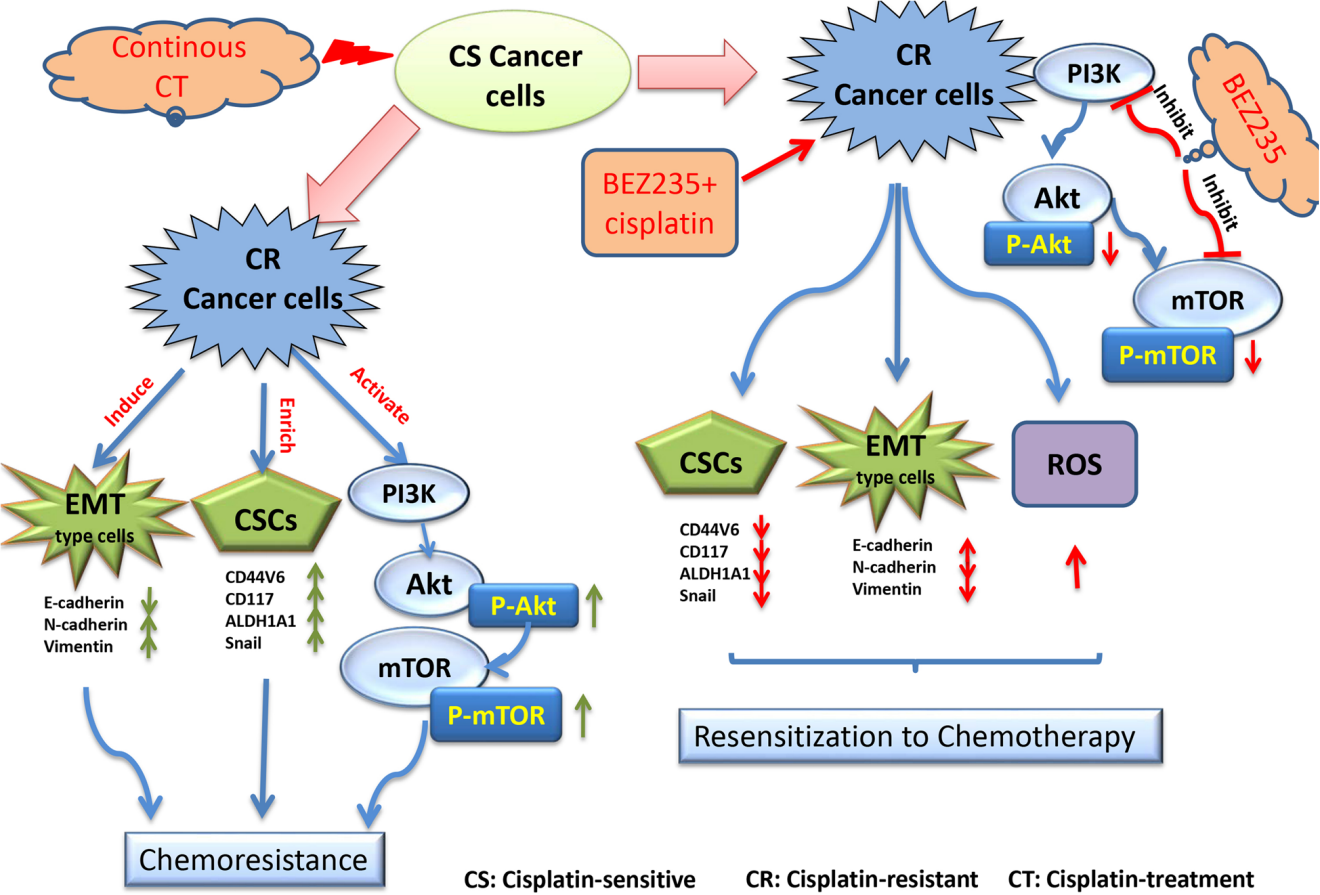
The potential mechanisms underlying EOC chemoresistance and the sensitizing effect of PI3K inhibition

Considering the close association of EMT and stemness with chemoresistance, we next investigated whether the PI3K/mTOR inhibition can suppress EMT and CSC marker expression of EOC-cis cells using western blot analysis. The results showed that the single treatment of BEZ235 could reverse the EMT of EOC-cis cells (decreased expression of E-cadherin and increased expressions of N-cadherin and Vimentin) (Fig. 4a). Furthermore, we found that single cisplatin treatment can enhance CSC marker expression of EOC-cis cells as evidenced by the increased protein expressions of CD44v6, CD117, ALDH1A1, and Snail **(**Fig. 4b**)**. In contrast, the combination treatment of BEZ235 and cisplatin not only reversed the EMT of EOC-cis cells but also abolished cisplatin-induced CSC marker expression **(**Fig. 4a and b**)**, suggesting that PI3K inhibition can sensitize EOC-cis cells to cisplatin through inhibiting EMT and CSC marker expression. The mechanisms underlying the sensitizing effect of PI3K/mTOR inhibition are summarized in Fig. 5.

## Discussion

Chemoresistance is a major clinical challenge for ovarian cancer therapy. The mechanisms underlying EOC chemoresistance remain elusive. Hence, elucidating the mechanisms underlying EOC chemoresistance might be expected to improve the poor prognosis. In this study, using the paired chemoresistant EOC cell lines, we demonstrate the role and association of PI3K/Akt/mTOR signalling, EMT, and CSC in EOC chemoresistance. A summary of our findings is shown in Fig. 5.

Our results demonstrate that cisplatin-resistant EOC cells are cross-resistant to chemotherapeutic drugs, including cisplatin, carboplatin, and paclitaxel, suggesting that common resistant mechanisms might exist within EOC cells. In addition, we also found that EOC cells possessing stem-cell or EMT characteristics were enriched, and the PI3K/Akt/mTOR pathway was activated in two cisplatin-resistant EOC lines. Furthermore, the dual inhibitor, BEZ235, was found to inhibit the cell viability of cisplatin-resistant EOC cells, reverse EMT, repress stem-cell-associated gene expression, and re-sensitize chemoresistant EOC cells to cisplatin.

In the first stage, we compared the characteristics of two pairs of EOC cell lines, the chemoresistant cell lines (A2780-cis and IGROV1-cis) and the corresponding chemosensitive cell lines (A2780 and IGROV1). The cisplatin-resistant cells showed multidrug-resistance, which might be developed by several mechanisms, such as decreased drug uptake, increased drug efflux, activation of detoxifying systems, activation of DNA repair mechanisms, evasion of drug-induced apoptosis [26].

In this study, the decreased E-cadherin and increased N-cadherin and Vimentin were found in cisplatin-resistant EOC cells compared with parental cells, which is consistent with the findings from other groups in EOC and lung cancer [27]. The increased expressions of EMT-related transcription factors, including Snail, Slug, Twist2, and Zeb2, at the gene level in A2780-cis cells further support our observation [28]. B Davidson, et al. [29] recently found that EMT markers-Vimentin and Zeb1 can be used as biomarkers for predicting poor response to chemotherapy in metastatic serous ovarian carcinoma effusions, further confirming the significance of EMT in EOC chemoresistance. Increasing findings indicate that EMT and CSC have a close link and share the same markers and properties [30]. Our results suggest that the acquisition of EMT in EOC cisplatin resistance is accompanied by an enhanced CSC marker expression, further proving the close association of EMT with CSCs.

The CSC theory offers a potential explanation for the relapse and resistance that occur in many tumors including ovarian cancer after therapy [31]. CSCs play very important roles in cancer metastasis and chemoresistance [32]. Targeting CSCs is promising and may overcome chemoresistance and lead to the cure of EOC [3]. In the current study, we found that two cisplatin-resistant EOC cell lines consistently showed increased ovarian cancer stem cell markers, indicating that CSCs may have an association with chemoresistance. Furthermore, the lower cell proliferation rate (quiescent status), increased G0/G1 phase, increased invasion and migration capability, were also observed in cisplatin-resistant cell lines, which are recognized characteristics maintained by CSCs [33–36].

Accumulating evidence demonstrates that PI3K/Akt/mTOR signalling, EMT, and CSCs play important roles in EOC progression, metastasis, and chemoresistance [3]. In this study, we found this pathway was activated in two cisplatin-resistant cell lines. Therefore, important questions arise as to whether there is a relationship between the activation of PI3K/Akt/mTOR signalling and EMT or enhanced CSC marker expression, and whether targeting this pathway could be effective to overcome EOC chemoresistance.

To deeply study the involvement of PI3K/Akt/mTOR signaling with acquisition of EMT and enhanced CSC marker expression in EOC chemoresistance, a dual inhibitor of PI3K/Akt/mTOR pathway, BEZ235, was selected for advancing our investigation. BEZ235, which targets both pan class I PI3K and mTOR, has been reported to inhibit cancer cell growth in vitro and in vivo [37, 38]. This dual inhibitor has been used in a Phase I/II clinical trial for advanced solid tumors and may be useful for EOC clinical trials in the future [39, 40]. In this study, an interesting finding is that the combination treatment or BEZ235 alone can reverse EMT and greatly reduce the expressions of CSC markers in cisplatin-resistant EOC cells compared with control or cisplatin alone, suggesting that the inhibition of PI3K/Akt/mTOR signaling can suppress EMT and CSC marker expression. Another interesting finding is that cisplatin can enhance CSC marker expression. This result is in line with the enhanced CSC marker expression found in EOC-cis cell lines as compared to the corresponding cisplatin-sensitive parental cells. Our results demonstrate that the inhibition of PI3K/Akt/mTOR pathway by BEZ235 confers a strong preferentially inhibitory effect on EMT and CSC. Also, more apoptosis was induced by combination therapy compared to single BEZ235 or cisplatin treatment, indicating that BEZ235 can increase chemosensitivity in cisplatin-resistant cells through inhibiting PI3K/Akt/mTOR signaling. Of note, BEZ235 has been demonstrated to be effective in targeting cells with CSC characteristics [19]. Dubrovska et al. found that the inhibition of PI3K/mTOR activity by BEZ235 led to a decrease in the population of CD133^+^/CD44^+^ prostate cancer progenitor cells in a mouse xenograft model [41]. It is well known that conventional chemotherapy drugs effectively target processes that cancer cells need to grow and divide, such as the ability to replicate DNA. CSCs are relatively resistant to chemotherapy mostly because of the quiescence and thus can be enriched by chemotherapy, which is evidenced by our data and findings from other researchers [10, 42]. These results support BEZ235 as a good candidate for CSC treatment.

Furthermore, we found that ROS and cell death caused by combination therapy were more significant than BEZ235 or cisplatin treatment alone in EOC-cis cell lines, indicating that inhibited oxidative stress is another possible mechanism underlying EOC cisplatin resistance and apoptosis [43]. ROS has an important implication in cancer chemoresistance and was reported to be associated with a developing cancer stemness [44, 45]. These studies further support our findings, i.e. increased ROS level and weakened CSC marker expression after combination treatment. It will be interesting to investigate the exact mechanisms of how ROS gets involved in EOC chemoresistance in the following study.

Finally, further investigation in high grade serous ovarian cancer is needed as an extension of this study to confirm the crosslink of PI3K/Akt/mTOR signaling with EMT and CSC. Findings from transgenic mice may be helpful for better understanding the relative importance of this pathway and underlying mechanisms in regulating EMT and stemness of ovarian cancer. Besides, cells sorted based on certain ovarian CSC surface markers may reveal the exact role of PI3K/Akt/mTOR signaling in regulating different ovarian CSCs at a single-cell level.

## Conclusions

All in all, we demonstrate for the first time that EOC chemoresistance is associated with PI3K/Akt/mTOR signaling, EMT, and CSCs, which contributes to cancer cell survival, migration, and invasion. Combination treatment of BEZ235 and chemotherapy can overcome EOC chemoresistance and holds promise for future ovarian cancer treatment.

## Additional files

Additional file 1: **Figure S1.** Proliferation and cell cycle analysis of EOC-cis and their parental cells. **(a)** The proliferation of A2780, A2780-cis, IGROV1, and IGROC1-cis was detected within 7 consecutive days. The proliferation rates of EOC-cis cell lines were significantly lower as compared to parental cell lines. (**b**) Cell cycle distribution of A2780, A2780-cis, IGROV1, and IGROV1-cis was detected using flow cytometry analysis. The proportion of cells in G0/G1 phase was significantly higher in EOC-cis cells compared with parental cells. The percentage of A2780-cis cells in G2/M phase was significantly lower than parent A2780 cells, while the percentage of IGROV1-cis cells in S phase was remarkably lower than IGROV1 cells. All data were expressed as mean ± SD and ^*^P < 0.05 versus control group (*n* = 3). (JPG 400 kb)

Additional file 2: **Figure S2.** Cross-resistance of EOC-cis cells to other important chemotherapeutic drugs. **(a)** A2780 and A2780-cis cells were treated with different concentrations of carboplatin and paclitaxel for 48 h. Cell viability was detected using cell proliferation assay. **(b)** IGROV1 and IGROV-1-cis cells were treated with different concentrations of carboplatin and paclitaxel for 48 h. Cell viability was detected using cell proliferation assay. All data were expressed as mean ± SD (*n* = 3). (JPG 603 kb)

Additional file 3: **Table S1.** Antibodies used for immunofluorescence (IF) staining and western blot (WB)**. Table S2.** Concentrations of cisplatin (μM) used in Fig. 1a (from left to right)**. Table S3.** IC50 values for EOC cell lines to different chemotherapeutic drugs at 48h. **Table S4.** The immunofluorescence staining scores for EMT markers in EOC cell lines. **Table S5.** The immunofluorescence staining results for CSC markers in EOC cell lines. (DOCX 20 kb)

## Data Availability

The datasets used and/or analyzed during the current study are available from the corresponding author on reasonable request.

## Abbreviations

Akt: Protein kinase B
ALDH1A1: Aldehyde dehydrogenase 1 family member A1
BEZ235: Dactolisib
CD117: Stem cell growth factor receptor
Cis: Cisplatin
C-PARP: Cleaved poly-ADP-ribose polymerase
CSC: Cancer stem cell
EMT: Epithelial-mesenchymal transition
EOC: Epithelial ovarian cancer
IC_50_: Half maximal inhibitory concentration
mTOR: Mammalian target of rapamycin
p-4EBP1: Phosphorylated 4E-binding protein 1
PI3K: Phosphoinositide 3-kinase
PTEN: Phosphatase and tensin homolog
qRT-PCR: Quantitative real-time PCR
ROS: Reactive oxygen species
