# Supplementary figures and images for: Inhibition of PI3K/Akt/mTOR signaling pathway alleviates ovarian cancer chemoresistance through reversing epithelial-mesenchymal transition and decreasing cancer stem cell marker expression
Source: BMC Cancer. 2019 Jun 24;19:618. doi: 10.1186/s12885-019-5824-9 (PMC6591840; doi:10.1186/s12885-019-5824-9)

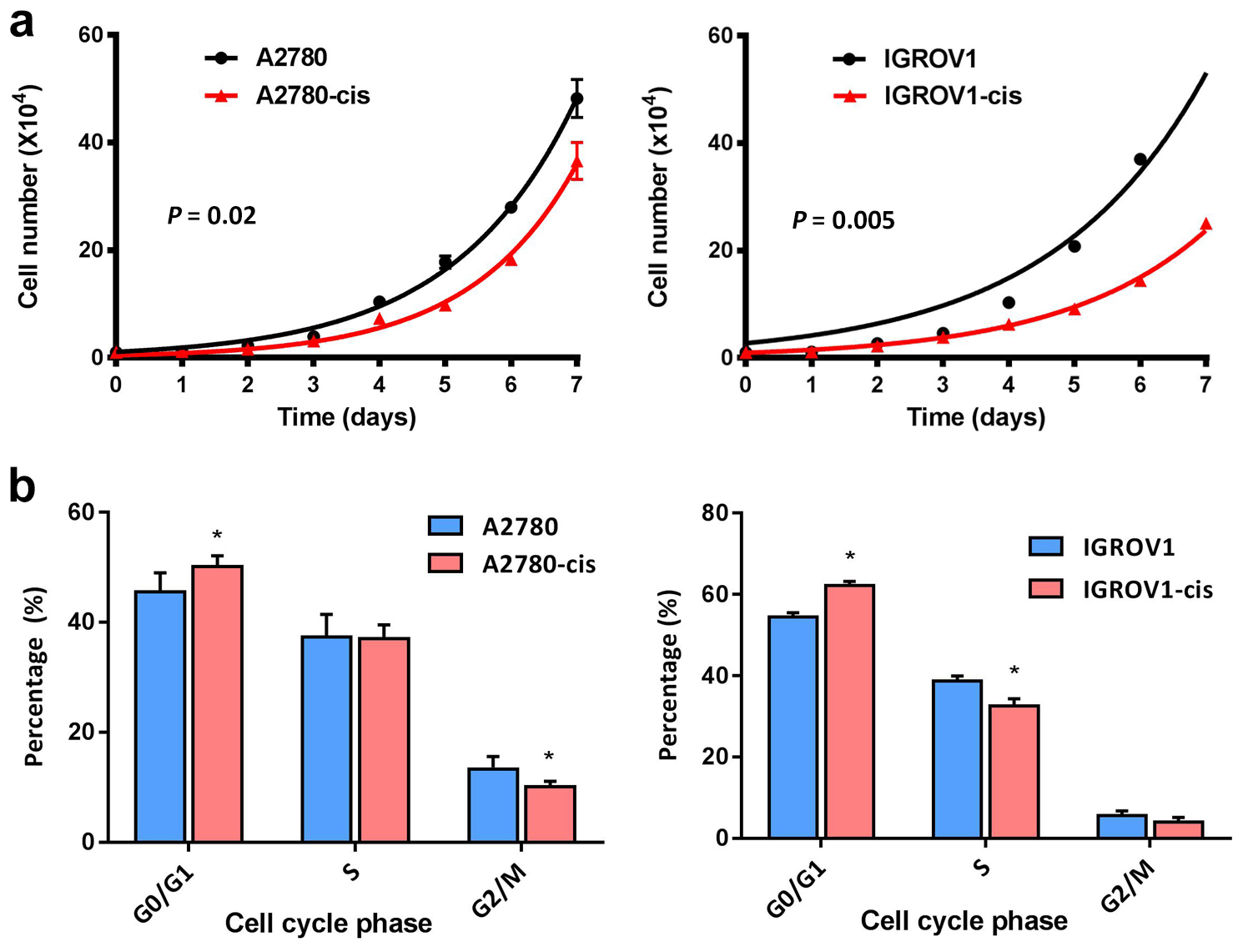

Supplement: Supplementary file 1 — Figure S1. Proliferation and cell cycle analysis of EOC-cis and their parental cells. (a) The proliferation of A2780, A2780-cis, IGROV1, and IGROC1-cis was detected within 7 consecutive days. The proliferation rates of EOC-cis cell lines were significantly lower as compared to parental cell lines. (b) Cell cycle distribution of A2780, A2780-cis, IGROV1, and IGROV1-cis was detected using flow cytometry analysis. The proportion of cells in G0/G1 phase was significantly higher in EOC-cis cells compared with parental cells. The percentage of A2780-cis cells in G2/M phase was significantly lower than parent A2780 cells, while the percentage of IGROV1-cis cells in S phase was remarkably lower than IGROV1 cells. All data were expressed as mean ± SD and *P < 0.05 versus control group (n = 3). (JPG 400 kb) [file 12885_2019_5824_MOESM1_ESM.jpg]

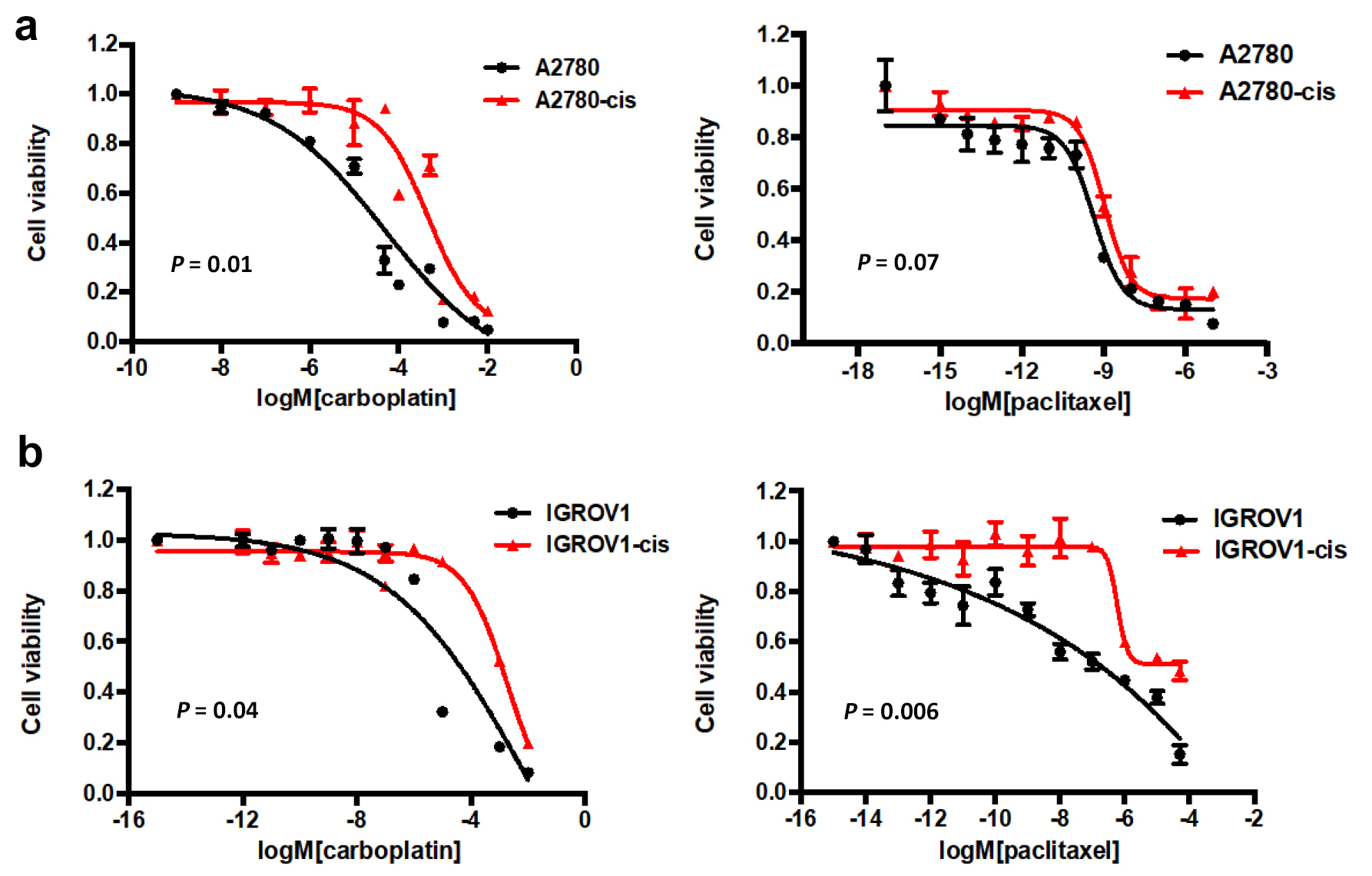

Supplement: Supplementary file 2 — Figure S2. Cross-resistance of EOC-cis cells to other important chemotherapeutic drugs. (a) A2780 and A2780-cis cells were treated with different concentrations of carboplatin and paclitaxel for 48 h. Cell viability was detected using cell proliferation assay. (b) IGROV1 and IGROV-1-cis cells were treated with different concentrations of carboplatin and paclitaxel for 48 h. Cell viability was detected using cell proliferation assay. All data were expressed as mean ± SD (n = 3). (JPG 603 kb) [file 12885_2019_5824_MOESM2_ESM.jpg]
