# Supplementary material for: Inhibition of PI3K/Akt/mTOR signaling pathway alleviates ovarian cancer chemoresistance through reversing epithelial-mesenchymal transition and decreasing cancer stem cell marker expression
Source: BMC Cancer. 2019 Jun 24;19:618. doi: 10.1186/s12885-019-5824-9 (PMC6591840; doi:10.1186/s12885-019-5824-9)
Supplement: Supplementary file 3 — Table S1. Antibodies used for immunofluorescence (IF) staining and western blot (WB). Table S2. Concentrations of cisplatin (μM) used in Fig. 1a (from left to right). Table S3. IC50 values for EOC cell lines to different chemotherapeutic drugs at 48h. Table S4. The immunofluorescence staining scores for EMT markers in EOC cell lines. Table S5. The immunofluorescence staining results for CSC markers in EOC cell lines. (DOCX 20 kb) [file 12885_2019_5824_MOESM3_ESM.docx]

**Table. S1.** **Antibodies used for immunofluorescence (IF) staining and western blot (WB).**

| **Antibody** | **Source** | **Type** | **Dilution ratio** | **Incubation time (min) and**  **temperature** |
| --- | --- | --- | --- | --- |
| Rabbit anti-human E-cadherin | Abcam | MAb | 1:2000 (WB)  1:200 (IF) | O/N, 4℃ |
| Rabbit anti-human N-cadherin | Abcam | PAb | 1:200 (WB)  1:200 (IF) | O/N, 4℃ |
| Mouse anti-human Vimentin | Santa Cruz | MAb | 1:1000 (WB)  1:100 (IF) | O/N, 4℃ |
| Mouse anti-human CD44v6 | Abcam | MAb | 1:1000 (WB)  1:100 (IF) | O/N, 4℃ |
| Rabbit anti-human CD117 | Abcam | PAb | 1:1000 (WB)  1:100 (IF) | O/N, 4℃ |
| Rabbit anti-human Snail | Abcam | PAb | 1:1000 (WB)  1:100 (IF) | O/N, 4℃ |
| Goat anti-human ALDH1A1 | Santa Cruz | PAb | 1:200 (WB, IF) | O/N, 4℃ |
| Rabbit anti-human Akt | Abcam | PAb | 1:1000 (WB) | O/N, 4℃ |
| Rabbit anti-human p-Akt1/2/3 | Santa Cruz | PAb | 1:1000 (WB) | O/N, 4℃ |
| Rabbit anti-human mTOR | Cell Signaling | PAb | 1:1000 (WB) | O/N, 4℃ |
| Rabbit anti-human p-mTOR | Cell Signaling | PAb | 1:1000 (WB) | O/N, 4℃ |
| Rabbit anti-human p-4EBP1 | Cell Signaling | MAb | 1: 1000 (WB)  1: 200 (IF, IHC) | O/N, 4℃ |
| Rabbit anti-active caspase 3 antibody | Abcam | PAb | 1:500 (WB) | O/N, 4℃ |
| Rabbit anti-human c-PARP | Abcam | MAb | 1:1000 (WB) | O/N, 4℃ |
| β-actin | Sigma-Aldrich | MAb | 1:3000 (WB) | O/N, 4℃ |
| Goat anti-rabbit IgG-HRP | Santa Cruz | IgG | 1:3000 (WB) | 60, RT |
| Goat anti-mouse IgG-HRP | Santa Cruz | IgG | 1:3000 (WB) | 60, RT |
| Donkey anti- Goat IgG-HRP | Santa Cruz | IgG | 1:3000 (WB) | 60, RT |
| Goat anti-mouse Alexa Fluor® 488 Dye Conjugate | Invitrogen | IgG | 1:1000 (IF) | 45, RT |
| Goat anti-rabbit Alexa Fluor® 488 Dye Conjugate | Invitrogen | IgG | 1:1000 (IF) | 45, RT |
| Donkey anti-goat Alexa Fluor® 488 Dye Conjugate | Invitrogen | IgG | 1:1000 (IF) | 45, RT |

**Abbreviation:** Mab, monoclonal antibody, O/N, overnight, PAb, polyclonal antibody, RT, room temperature.

**Table. S2. Concentrations of cisplatin (μM) used in Fig.1a (from left to right).**

| **Cell line** | **1** | **2** | **3** | **4** | **5** | **6** | **7** | **8** | **9** | **10** | **11** | **12** |
| --- | --- | --- | --- | --- | --- | --- | --- | --- | --- | --- | --- | --- |
| A2780 | 0.01 | 0.1 | 0.5 | 1 | 2 | 5 | 10 | 20 | 50 | 100 | 500 | 1000 |
| IGROV1 | 10^-9^ | 10^-7^ | 10^-6^ | 10^-5^ | 10^-4^ | 10^-3^ | 0.01 | 0.1 | 1 | 10 | 100 | 1000 |

**Table. S3. IC_50_ values for EOC cell lines to different chemotherapeutic drugs at 48h.**

| **IC_50_** | **A2780** | **A2780-cis** | **Fold change** | **IGROV1** | **IGROV1-cis** | **Fold change** |
| --- | --- | --- | --- | --- | --- | --- |
| Cisplatin | 2.5µM | 25.7µM | 10.3 | 4.0µM | 135 µM | 33.7 |
| Carboplatin | 29.5µM | 468 µM | 15.9 | 46.7µM | 1580 µM | 33.8 |
| Paclitaxel | 0.4nM | 1.4 nM | 3.5 | 0.3 µM | 46.8 µM | 156 |

**Notes:** IC_50_ values of the three chemotherapeutic drugs (cisplatin, carboplatin, and paclitaxel) on EOC-cis cell lines were significantly higher compared to EOC-control cell lines (*P < 0.05*).

**Table. S4.** **The immunofluorescence staining scores for EMT markers in EOC cell lines.**

| **Biomarker** | **A2780** | **A2780-cis** | **IGROV1** | **IGROV1-cis** |
| --- | --- | --- | --- | --- |
| E-Cadherin | 3 | 0 | 2 | 0 |
| N-Cadherin | 0 | 2 | 1 | 3 |
| Vimentin | 1 | 3 | 1 | 3 |

**Note:** “0“ represents negative staining, “1”, “2” , “3” represent weak staining, moderate staining, and strong staining, respectively.

**Table. S5. The immunofluorescence staining results for CSC markers in EOC cell lines**

| **Biomarker** | **A2780** | **A2780-cis** | **IGROV1** | **IGROV1-cis** |
| --- | --- | --- | --- | --- |
| CD44v6 | 1 | 3 | 1 | 2 |
| CD117 | 0 | 3 | 2 | 3 |
| ALDH1A1 | 2 | 3 | 0 | 3 |
| Snail | 1 | 3 | 1 | 3 |

**Note:** “0“ represents negative staining, “1”, “2” , “3” represent weak staining, moderate staining, and strong staining, respectively.
